# Maternity waiting homes as component of birth preparedness and complication readiness for rural women in hard-to-reach areas in Ethiopia

**DOI:** 10.1186/s12978-021-01086-y

**Published:** 2021-02-02

**Authors:** Mekdes Kondale Gurara, Jean-Pierre Van Geertruyden, Befikadu Tariku Gutema, Veerle Draulans, Yves Jacquemyn

**Affiliations:** 1 Department of Public Health, College of Medicine and Health Sciences, Arba Minch University, P.O. Box 21, Arba Minch, Ethiopia; 2 Global Health Institute, Faculty of Medicine & Health Sciences, University of Antwerp, Wilrijk, Belgium; 3 Faculty of Social Sciences, Centre for Sociological Research, KU Leuven, Leuven, Belgium; 4 Department of Obstetrics and Gynecology, Antwerp University Hospital, Edegem, Belgium

**Keywords:** Maternity waiting home, Ethiopia, Birth preparedness plan, Access, Logistic barriers, Low-income, Pregnancy, Maternal health

## Abstract

**Background:**

In rural areas of Ethiopia, 57% of births occur at home without the assistance of skilled birth attendants, geographical inaccessibility being one of the main factors that hinder skilled birth attendance. Establishment of maternity waiting homes (MWH) is part of a strategy to improve access to skilled care by bringing pregnant women physically close to health facilities. This study assessed barriers to MWHs in Arba Minch Zuria District, Southern Ethiopia.

**Methods:**

A community-based cross-sectional study was undertaken from February 01 to 28, 2019. Study participants were selected by computer-generated random numbers from a list of women who gave birth from 2017 to 2018 in Arba Minch Health and Demographic Surveillance System site. Data were collected using a pre-tested and interviewer-administered questionnaire. Stata software version-15 was used for data management and analysis, and variables with *p*-values ≤ 0.2 in bivariate analysis were considered for multivariable logistic regression analysis. Level of statistical significance was declared at a *p*-value < 0.05. Qualitative data were analyzed manually based on thematic areas.

**Results:**

MWH utilization was found to be 8.4%. Wealth index (lowest wealth quintile aOR 7.3; 95% CI 1.2, 42), decisions made jointly with male partners (husbands) for obstetric emergencies (aOR 3.6; 95% CI 1.0, 12), birth preparedness plan practice (aOR 6.5; 95% CI 2.3, 18.2), complications in previous childbirth (aOR 3; 95% 1.0, 9), history of previous institutional childbirth (aOR 12; 95% CI 3.8, 40), residence in areas within two hours walking distance to the nearest health facility (aOR 3.3; 95% CI: 1.4, 7.7), and ease of access to transport in obstetric emergencies (aOR 8.8; 95% CI: 3.9, 19) were factors that showed significant associations with MWH utilization.

**Conclusions:**

A low proportion of women has ever used MWHs in the study area. To increase MWH utilization, promoting birth preparedness practices, incorporating MWH as part of a personalized birth plan, improving access to health institutions for women living far away and upgrading existing MWHs are highly recommended.

## Plain english summary

One of the ways to reduce maternal and neonatal deaths is to ensure that there is a skilled birth attendant (SBA) at every child-birth. However, in rural Ethiopia, more than half of all births occur at home without the help of SBAs. In remote and hard to reach areas, geographical and logistical barriers are among the main factors hindering skilled birth attendance. Maternity waiting homes (MWH) have been implemented in rural districts of Ethiopia with the aim to address these deterrents. The purpose of this study was to assess the barriers to MWHs use in the rural district of southern Ethiopia.

Face-to-face interviews were conducted among 807 women who gave birth two years before the study period. A structured questionnaire was used to collect quantitative data. We have also conducted focus group discussions to get a better insight into the barriers to MWHs utilization.

Overall, we found a low MWH utilization; women who prepared for birth and its possible complication had higher rates of MWH use. Moreover, women in the low wealth quintile, who made decisions jointly with their partners, who had a history of previous institutional childbirth, who were residing within two hours walking distance, and had the ease of access to transport were more likely to use the existing MWH services. To increase MWH utilization with the aim to improve access to SBA, promoting birth preparedness practices, incorporating MWH as part of a personalized birth plan, promoting MWHs for women living far away, and upgrading existing MWHs are highly recommended.

## Background

Maternal mortality continues to be a public health problem globally despite the existence of effective interventions to curb it. In 2017, an estimated 295,000 women died from preventable obstetric complications worldwide, 94% of the deaths occurring in low-income countries. The sub-Saharan region accounted for 66% of the deaths, with 59% occurring in Ethiopia alone, a share that was among the top ten by country in the world [1]. Moreover, for every woman who dies of pregnancy-related complications, about 20–30 others experience morbidity globally, and the burden is adjudged to be highest in low income-countries [1, 2].

Ensuring skilled care at delivery, backed up by easy and available transport in case of emergency referral, is vital in reducing maternal morbidity and mortality [3]. Yet, the lack of timely planning for skilled care for normal births and inadequate preparations for obstetric complications is common in low-income countries, especially in Ethiopia where more than 50% of all births are not assisted by skilled providers [4]. It has been proven that making a birth plan during pregnancy reduces delay in obtaining care and promotes the timely use of emergency obstetric care [5–8]. The major components of birth preparedness include: selecting a desired place of birth, identifying a skilled provider and making the necessary arrangements to receive skilled care for normal births, and preparing for prompt action in the event of an obstetric emergency [5].

Yet, transportation issues, such as the poor condition of local roads and the distance of health institutions (HIs) from the homes of pregnant women, appear to be important factors influencing access to timely obstetric care and may lead to poor pregnancy outcomes [9]. To overcome the major logistic barriers in low-income countries, the establishment of maternity waiting homes (MWHs) at or around HIs has been widely adopted in addition to the common birth preparedness plans. MWHs are homes or shelters built nearby or in the compound of HIs, where pregnant mothers can lodge so that, when they go into labor, they can be quickly transferred to the facilities for safe delivery [5, 10–13]. Regarding the contributions of the homes, several studies have reported that MWHs improve attendance to antenatal and postnatal care and reduce the odds of homebirth [12, 14–17]. However, as MWHs have not been considered as one component of a personalized birth plan, there is still a very huge proportion of homebirths in rural and hard-to-reach areas [18]. Poor quality of care at both the MWHs and the adjacent facilities, structural barriers, costs associated with transportation, and psychosocial aspects of the care also result in limited use of the existing MWH services [19–24].

However, to the best of the researchers’ knowledge, there is no study in the literature which addresses the association between MWH use and birth preparedness plan in Ethiopia. This study, therefore, considers birth preparedness practice as a factor which may affect stay at MWHs. Although MWH intervention has been promoted in the study area to decrease delays in access to maternal care, previous studies have reported that only 9.7% of the women completed the continuum of care [25] and 18.5% of births were assisted by skilled birth attendants [22, 26], with about 30% prevalence of birth preparedness plans in 2017 [27]. Evaluation of contextually related barriers and constraints to access to MWH services using mixed methods design may improve maternal health. Therefore, this study aimed to explore the barriers to the use of MWHs, taking into account the social-cultural and economic factors of the participants, birth preparedness practices, and geographic specifics of the setting.

## Methods

### Study area and setting

The study was conducted at Arba Minch Health Demographic Surveillance site (HDSS), which is located at Arba Minch Zuria District of Gamo Zone, Southern Ethiopia. The district has a total of 29 kebele (the smallest administrative structure in Ethiopia): 2 semi-urban and 27 rural. Out of the 29 kebele in the district, 9 (eight rural and one semi-urban) were selected randomly based on their geo-climatic zone (high-, mid- or lowland). The surveillance site uses an Open Data Kit (ODK) tablet computer-based application for data collection with updates conducted biannually. Since the HDSS establishment in 2009, there has been continuous recording of vital events and utilization of health services, including verbal autopsy for the cause of death. According to the 2007 Ethiopian national census, the estimated projected total population of the district for 2017 was 195,858, of which 50% were female. There are seven health centers in the surveillance site; six of the health centers are attached each with an MWH as a physically separate room. At the health center level, basic emergency obstetric care services are offered. The health center is typically staffed by health officers, midwives, and nurses and when they are faced with obstetric complications, they refer the women to Arba Minch general hospital where comprehensive emergency obstetric care is offered.

### Study design and period

A community-based cross-sectional study using mixed methods design was conducted between February 1 and 28, 2019.

### Population of the study

The source population for this study comprised of all women in Arba Minch Zuria District who had given birth within two years to the beginning of the survey. The study population was women in the nine kebele of Arba Minch HDSS site who had given birth within two years to the survey. During data collection, women who were seriously ill or unable to give information were excluded from the study.

### Sample size determination and sampling technique

A single population proportion [28] formula was used to estimate the minimum sample size required for the study, with an estimated level of MWH utilization of 31.5% adopted from a previous study which was conducted in Zambia [16], a maximum tolerable error of 5%, and a value of standard normal distribution (z-statistic) of 1.96 at 95% confidence level. The calculated sample size using the above assumptions was 663. In addition, the sample size for the barriers to MWH use (women’s occupation, household index, and travel time to obstetric care facility) [29] was computed yet the result yielded a smaller sample size than the level of utilization. An additional 10% from the estimated sample size was considered to compensate for potential non-responses and multiplying the design effect by two, and the final sample size for the study was 814.

A sampling frame, a list of 2187 women who gave birth between 2017 and 2018, was obtained from the HDSS database and used to select the study unit. The required sample size was proportionally allocated to each kebele based on the total number of eligible women per kebele. The eligible women for interviews were then selected using computer generated random numbers. For qualitative data, purposive sampling technique was used to select focus group discussants. Community health extension workers were contacted first to recruit women who used MWHs (users) and those who did not (non-users). One of the reasons for using the qualitative approach in this particular study was to provide a more in-depth picture of the situation, as qualitative data would reflect the cultural beliefs and practices that need to be considered for MWH service improvement in the future. Four focus group discussions (six to eight participants per group) were conducted among MWH users (2) and MWH non-users (2).

### Data collection methods and procedures

A structured interviewer-administered questionnaire was developed after reviewing the relevant literature in the field (see Additional file 1). Socio-demographic information, obstetric and reproductive health information, and data related to MWHs awareness and utilization were collected using ODK (smart phone or tablet computer-based application for data collection) through face-to-face interviews. Ten HDSS data collectors and 6 site supervisors working in the district participated in the data collection. Individual and household identity numbers (every household/individual has a unique identification number in the surveillance site) which were generated along with the list of eligible women from the HDSS database were used to access houses of the sampled women.

For the qualitative data, semi-structured open-ended focus group discussion (FGD) guides were developed to facilitate the discussions (see Additional file 2). The principal author moderated the discussion in the presence of a facilitator from each of the kebele. Group discussions were conducted in health post halls. Idea saturation was used to determine the number of FGDs and each discussion was tape-recorded in order not to forget any issue discussed.

### Data quality management

Two days of intensive training was given to the data collectors and supervisors by the study team with a particular emphasis on the objective of the study and the content of the questionnaire. The quantitative questionnaire used to obtain the data was initially prepared in English and translated to Amharic language by an expert in the language, and finally translated back to English to check its consistency with the original meanings. Prior to data collection, pre-testing was conducted outside the study area on 5% of the sample and some important improvements were made on the content of the questions. The subjects were interviewed in separate private places to minimize social desirability bias. Since we used a mobile Open Data Kit application to record responses to structured interviews with participants, data entry related errors were nil.

### Data analysis

Participants’ responses which were recorded in mobile Open Data Kit applicationwas exported to Stata version 15 software package for further management and analysis. Descriptive statistics were used to describe the level of utilization of MWH services and explanatory variables. The findings were presented in narration, tables and graphs as frequencies, proportions, and means, with their corresponding standard deviations. The principal component analysis (PCA) was used to construct the socioeconomic status (wealth index) of the households where the study participants lived. The questions were adapted from the Ethiopian Demographic Health Survey 2016 [30], which included information on household ownership of durable assets, house and livestock, housing characteristics, and access to utilities and infrastructure. All variables that supposedly measure household wealth were coded into binary variables (0/1). Wealth quintiles (five equal groups) were then constructed based on weights for each asset with the 1st quintile representing the bottom (poorest) households and the 5th quintile representing the top (richest) households in the sample.

Binary logistic regression analysis was performed to identify factors affecting the use of MWH services. Primarily, the association between each explanatory variable and the dependent variable was checked using bivariate logistic regression analysis. Then, explanatory variables which showed association at the bivariate level with p-values of less than 0.25 were considered using multivariable logistic regression analysis. Association between the dependent variable and the explanatory variables was reported using an adjusted odds ratio at a 95%-confidence interval. Finally, statistical significant association was declared at a *p*-value of ≤ 0.05. With regard to multi-collinearity, Variance inflation factor was calculated to check the collinearity between independent variables and we found the maximum value of 2.16 for all explanatory variables included in the multivariable logistic regression. For the purpose of analysis, the recorded audio discussion was transcribed verbatim and categorized according to the themes that emerged, and narrative quotes have been presented to support the quantitative findings.

### Definition and operationalization of variables

The dependent variable for this study was the use of MWH services. Those mothers who had stayed in MWHs during their last pregnancy were considered as MWH users while those that had never used MWH were regarded as non-users. The explanatory variables included socio-demographic variables, obstetric-related variables, MWH awareness, accessibility to health institutions’, and birth preparedness and complication readiness (BPCR) practice.

To determine their level of preparedness, the study subjects were asked about the five basic components of BPCR: whether, for their last pregnancy, they saved up for their delivery, arranged ahead for transport, and identified in advance a place of delivery, a skilled attendant and a blood donor; and a woman was considered as “prepared” if she practiced at least three of the five components [31–33].

## Results

### Socio-demographic characteristics of the study participants

A total of 807 women, who had recently given birth, participated in this study. The mean age of the participants was 31.9 years (± 5.1). The majority of study participants were from the rural area (91%), housewives (85.9%), married (99.6%) and had no formal schooling (70%) (Table 1). Four FGDs were conducted with 29 women: 15 women who admitted to HIs via MWHs and 14 non-users (either directly admitted to HIs to give birth or gave birth at home or en route to HIs.)

**Table 1:** Socio-demographic characteristics of the participants in Arba Minch zuria district, 2019 (n = 807)

Variables	Categories	Frequency	Percent
Age category	15–24	40	5.0
	24–35	506	62.7
	35–48	261	32.3
Residency	Semi-Urban	73	9.1
	Rural	734	90.9
Occupation	Housewife	693	85.9
	Daily laborer	8	7.0
	Farmer	37	32.5
	Government employee	2	1.8
	Private business	47	41.2
	Merchant	15	13.2
	Other	5	4.4
Ethnicity	Gamo	687	85.1
	Wolayta	14	1.7
	Zeyse	93	11.5
	Other^a^	13	1.6
Religion	Orthodox	244	30.2
	Protestant	546	67.7
	Traditional	15	1.9
	Jehovah's Witness	2	0.2
Marital Status	Married	804	99.6
	Separated	2	0.3
	Single	1	0.1
Educational status^b^ of the mothers	Illiterate	565	70.0
	Read and write	3	0.4
	Elementary & Primary	200	24.8
	Secondary & preparatory	32	4.0
	Above grade 12	7	0.9
Additional job than being housewife	No	693	85.8
	Yes	114	14
Education status2 of the Husbands (n = 804)	Illiterate	489	60.8
	Read and write	10	1.2
	Elementary & Primary	239	29.7
	Secondary & preparatory	44	5.5
	Above grade 12	22	2.7
Husbands’ occupation (n = 804)	Daily laborer	71	8.8
	Farmer	637	79.2
	Government employee	21	2.6
	Merchant	36	4.5
	Private	15	1.9
	Other^c^	24	3.0
Wealth quintile*	1st quantile	163	20.2
	2nd quantile	160	19.8
	3rd quantile	162	20.1
	4th quantile	161	20.0
	5th quantile	161	20.0

^a^(2 Amhara, 7 Oromo, 1 Koyira 1 Konso, 1 Gurage, 1 Ganjule)

^b^(Educational status: Elementary & Primary is from grade 1–8, and Secondary and preparatory is grade from 9–12)

^c^(4 Pastor, 6 Jobless, 3 Driver, 4 Broker, 5 Student and 2 Retired)

*(wealth quantile: wealth quantile has been constructed based on the principal component analysis approach. The questions were adopted from the Ethiopian Demographic Health Survey 2016 and included household ownership of assets, house and livestock, housing characteristics, and access to utilities and infrastructure, and then the household's possession of the assets or materials was coded into binary variables (No = 0/Yes = 1). Then households’ wealth quintiles (five equal groups) were constructed based on the weights for each asset from the 1st quintile (poorest) to 5th quantile (richest) in the study population.)

### Obstetric characteristics and birth preparedness plan

Nearly all (98.6%) participants were multiparous, 706 of the women (87.48%) had antenatal visits, three out of the five mothers (62.4%) had given birth without the assistance of skilled providers, and only one in five (21.9%) women had prepared for birth and its possible complications during their last pregnancy (Table 2).

**Table 2:** Obstetric characteristics, birth preparedness plan practice and use of MWHs among the participants in Arba Minch Zuria district 2019 (n = 807)

Variables	Categories	Frequency	Percent
Age at first marriage	< 18	189	23.4
	≥18	618	76.6
Age at first pregnancy	< 18	62	7.7
	≤18	745	92.3
Parity	Primi-Para (1)	11	1.4
	Multi-para (2–4)	417	51.7
	Grand multipara (≥ 5)	379	46.9
Pregnant during study period	No	751	93.2
	Yes	55	6.8
History of neonatal death	No	735	91.1
	Yes	72	8.9
Family size^a^	≤4	494	61.2
	5–6	243	30.1
	≥7	70	8.7
History of family planning use	Currently using	393	48.7
	Never used	244	30.2
	Used in the past	170	21.1
BPCR plan^b^	Not prepared	636	78.8
	Prepared	171	21.2
ANC visits	No visit	101	12.5
	ANC 1–3	333	41.3
	ANC 4 +	373	46.2
Place of ANC	Health centers	199	28.2
	Health posts	501	71.0
	Hospital	6	0.8
Decision maker for obstetric care seeking	Husband/partner	138	17.1
	Jointly	634	78.6
	Respondent	35	4.3
Place of last birth	Home	504	62.0
	Health facility	303	38.0
Mode of delivery	Cesarean delivery	21	2.6
	Vaginal delivery	786	97.4
Previous complications during childbirth	No	766	94.9
	Yes	41	5.1
Seek treatment for the complications from health facilities	No	7	17.1
	Yes	34	82.9
Travel time to the nearest health institution	< 2 h	658	82
	> 2 h	149	18
Difficulty to get emergency transport	Difficult	354	43.9
	Easy	130	16.1
	Very difficult	323	40.0
MWH Awareness	No	278	34.5
	Yes	529	65.5
Sources of information	Community leaders	68	12.8
	Health extension workers	324	61.2
	Health facility staff	124	23.4
	Other	14	2.6
MWH location	No	288	35.7
	Yes	519	64.3
MWH stay (users)	No	739	91.6
	Yes	68	8.4

^a^Number of individuals in a household

^b^BPCR plan: To measure birth plan practices of the study subjects, participants were asked the five basic components of birth plan practice based on their importance (planned to save money to the childbirth, planned to arrange transport, identified place to give birth, identified skilled attendant and identified blood donor for their last pregnancy) and a woman was considered as “prepared” for birth and its complication if she reported at least three of the five basic components

### Utilization of the existing MWHs

Nearly two out of three (65.55%) participants have heard about the existence of MWHs at nearby healthcare facilities. The main source of this information was community health extension workers (61%). Only 68 (8.43%) of the participants used MWHs during their last pregnancy. Out of the 68 women, transportation problem (46women (67%)), absence of food catering at the MWHs (51 women (75%)), and poor availability of utensils (11 women (16%)) were the main challenges they faced during their stay (Table 3).

**Table 3:** MWH users experience in Arba Minch Zuria district in 2019 (n = 68)

Variables	Categories	Freq	Percent
Referral to MWH	Health centers	5	7.4
	Health extension workers	62	91.1
	Decided by myself	1	1.5
Length of stay (number of days)	< = 5 days	64	94.1
	> 5 days	4	5.9
Decision to stay at MHW	Husband	6	8.8
	Jointly	58	85.3
	Health care providers	3	4.4
	Respondent	1	1.5
Companionship during MWH stay	Husband	59	86.8
	Mother	2	2.9
	Neighbors	3	4.4
	Relatives	4	5.9
Financial support during MWH stay	Husband	61	89.7
	Myself	1	1.5
	Neighbors	2	2.9
	Relatives	4	5.9
Social support during MWH stay	Husband	51	75
	Neighbors	6	8.8
	Relatives	11	16.2

Corroborating the quantitative findings (Table 4), the FGD discussants highlighted that substandard service at the MWHs and adjacent health centers are among the reasons most pregnant women do not make use of the existing MWHs, as illustrated below:

One of the discussants, a 28-year-old lady, said: I decided to stay in this home [MWH] and I was escorted by two of my relatives. The room had no partition for changing your clothes, was small, and couldn’t occupy more than three people at a time. How do you protect your privacy in this room with no space for the ones that accompanied you? Guess what will happen? You would rather prefer not to come here. [Age 28, MWH user].

**Table 4:** Themes and illustrative quotes of participants’ responses during FGDs about the facilitators and barriers to use MWH services in Arba Minch zuria district, 2019

Themes:1 Facilitators to use MWH services	
Subthemes	Illustrative quotes
1a. Low socioeconomic status	*I wanted to go to [the] MWH, not because I was told about the standards of care there, but because I couldn't afford the payment for transportation, even I didn’t have alternative transportation means in case something [unforeseen] happens during childbirth at home. Instead, I preferred to be close to the facility with professional care*. [Age 33, MWH user]
1b. Negative childbirth experiences	*The very reason I decided to stay in [the] MWH was my shocking experience during my second childbirth. …I was with [a] traditional birth attendant. For some reason I don’t know, I had started bleeding shortly after the child was born and the blood was a lot that the traditional birth attendant couldn’t do anything… my village is quite far, walked long to get there [health center], and I was between life and death when I arrived there. If I had not received professional care at that time I would not be alive.* [Age 28, MWH user]
1c. Cultural sensitivity of the services	*MWH was built based on our culture, I can say it is exactly the same as my home [home away from home], and it is nearby the health centers where you can get professional care and [be] treated immediately if difficulties occur during childbirth. Also, it is an ideal place where pregnant women could share information among themselves. However, improving the absence of food catering and providing basic supplies for the duration of stay and basic sanitation would possibly satisfy users and increase future uptake of the service*. [Age 30, MWH user]
Subtheme 2 Barriers to use MWH services	
2a. Substandard of care and services at MWHs	*One of the discussants, a 28-year-old lady, said: I decided to stay in this home [MWH] and I was escorted by two of my relatives. The room had no partition for changing your clothes, was small, and couldn’t occupy more than three people at a time. How do you protect your privacy in this room with no space for the ones that accompanied you? Guess what will happen? You would rather prefer not to come here.* [Age 28, MWH user] *There used to be food catering in [MWHs] during the time the community used to contribute for the service. But that stopped, so I brought my own food, but there was no money to continue buying food after two days. I didn’t have other options than to return back home. I also remember there were pregnant women who wanted to prepare their own food, but they couldn't because there were no designated area and utensils to cook. So, they had to buy food.* [Age 32, MWH user] *For my last-child, as the health facility is quite far away from my residence and the time was rainy season, I had stayed in the MWH before delivery. It is good to have this kind of home where pregnant women can rest but I had noticed that cleanliness at the home [MWH] was poor and no one was responsible for it*. [Age 24, MWH user]
2b. Negative perception by healthcare providers	*Even though we have good health care providers who make us feel as if we are at our home [health center] … I'm sorry to say that… there are few who discourage us from using [the] MWH. Literally, they encourage you to return back to your home as long as you have someone who would escort you to the facility when active labor begins.* [Age 32, MWH non-user]
2c. Lack of women’s autonomy	*I knew about the existence of MWHs in my catchment [area] but I had to request my husband’s permission to use it. I and my husband made [a] joint decision about that during my last pregnancy; it would have been unlikely to use [the] MWH without his consent.*[Age 20, MWH user]
3d. Absence of someone to care for children at home	*As you leave your home, someone has to take care of all the responsibilities you have at home, including the caring and rearing roles*. [Age 20, MWH user]

The MWH users also frequently mentioned that the absence of food catering and the poor quality of care at the MWHs are the prime reasons why most women return to the homes after a few days at the MWHs, as illustrated below:

There used to be food catering in [MWHs] during the time the community used to contribute for the service. But that stopped, so I brought my own food, but there was no money to continue buying food after two days. I didn’t have other options than to return back home. I also remember there were pregnant women who wanted to prepare their own food, but they couldn't because there were no designated area and utensils to cook. So, they had to buy food. [Age 32, MWH user].

For my last-child, as the health facility is quite far away from my residence and the time was rainy season, I had stayed in the MWH before delivery. It is good to have this kind of home where pregnant women can rest but I had noticed that cleanliness at the home [MWH] was poor and no one was responsible for it. [Age 24, MWH user].

Another issue raised by most of the users was the attitudes of the providers towards the expectant mothers, which most likely influence the uptake and utilization of the MWHs, as illustrated below:

Even though we have good health care providers who make us feel at home [health center] … I'm sorry to say that… there are few who discourage us from using [the] MWH. Literally, they encourage you to return back to your home as long as you have someone who would escort you to the facility when active labor begins. [Age 32, MWH non-user].

The FGD discussants most frequently mentioned previous childbirth complications, poor transport alternatives and long-distance travels through mountainous terrains to the facilities, and their husbands’ consent as factors that make them use the MWHs, as illustrated below:

I wanted to go to [the] MWH, not because I was told about the standards of MWH services, but because I couldn't afford the payment for transportation, even I didn’t have alternative transportation means in case something [unforeseen] happens during childbirth at home. Instead, I preferred to be close to the facility with professional care. [Age 33, MWH user].

I knew about the existence of MWHs in my catchment [area] but I had to request my husband’s permission to use it. I and my husband made [a] joint decision about that during my last pregnancy; it would have been unlikely to use [the] MWH without his consent. As you leave your home, someone has to take care of all the responsibilities you have at home, including the caring and rearing roles. [Age 20, MWH user].

The very reason I decided to stay in [the] MWH was my shocking experience during my second childbirth. …I was with [a] traditional birth attendant [TBA]. For some reason I don’t know, I had started bleeding shortly after the child was born and the blood was a lot that the TBA couldn’t do anything… my village is quite far, walked long to get there [health center], and I was between life and death when I arrived there. If I had not received professional care at that time I would [not] be alive. [Age 28, MWH user].

MWH was built based on our culture, I can say it is exactly the same as my home [home away from home], and it is nearby the HIs where you can get professional care and [be] treated immediately if difficulties occur during childbirth. Also, it is an ideal place where pregnant women could share information among themselves. However, improving the absence of food catering and providing basic supplies for the duration of stay and basic sanitation would possibly satisfy users and increase future uptake of the service. [Age 30, MWH user].

### Factors affecting MWH utilization

The women’s economic status, decisions made jointly with male partners (husbands) for obstetric emergency, history of previous institutional childbirth, birth preparedness plan practice, history of previous childbirth complications, < 2hrs travel distance to the nearest HI, and ease of access to transport in case of obstetric emergency were independent factors significantly associated with MWH use after adjusting for the effects of other variables**.**

Accordingly, those women who live in households that fell within the 1st (AOR = 7.3; 95%CI: 1.2, 42) and 2nd wealth quintiles (AOR = 8.9; 95%CI: 1.8, 42) were more likely to use MWHs than those women grouped under the 5th quintile. Those women who made decisions jointly with their husbands to seek obstetric care (AOR = 3.6; 95%CI: 1.1, 12) had 3.6 times higher odds of using MWHs than those women whose husbands alone took the decisions. Concerning BPCR practice, those women who had prepared for birth and its possible complications were 6.5 (AOR = 6.5; 95%CI: 2.3, 18) times more likely to use MWHs than those women who did not prepare for birth and its possible complications.

Compared to those mothers who had no history of institutional childbirth, the odds of staying in MWHs by those women who have a history of previous health institutional childbirth is 12-times higher (AOR = 12; 95%CI: 3.8, 40). Those mothers who experienced complications during their previous childbirth were 3 times (AOR = 3; 95%CI: 1.0, 9) more likely to use MWHs during the following pregnancy compared with those mothers who had no such experience.

Those women residing in areas within 2 h of walking distance to the nearest HI were 3.3 times (AOR = 3.3; 95%CI: 1.4, 7.7) more likely to stay in MWHs compared to those women residing in areas farther than 2 h of walking distance. Similarly, those women who had easy access to transport in case of obstetric emergency were 8.8 times (AOR = 8.8; 95%CI: 3.9, 19.8) more likely to stay in MWHs compared to those women who had difficulty in accessing transport for obstetric emergency (Table 5).

**Table 5:** Factors associated with MWHs use in Arba Minch zuria district, 2019 (n = 807)

Variable	Categories	Used MWH		Unadjusted OR (95% CI)	Adjusted^b^ OR (95% CI)
		No	Yes		
Mothers’ educational status	No formal education	542	26	1	1
	Formal education	197	42	4.4 (2.6, 7.4)	0.6 (0.2,1.3)
Wealth index	1st quantile	160	3	0.09 (0.03,0.28)	7.3 (1.2,42)*
	2nd quantile	154	6	0.18 (0.07,0.4)	8.9 (1.8, 42)*
	3rd quantile	156	6	0.18 (0.07,0.4)	3.9 (0.8,18)
	4th quantile	137	24	0.80 (0.4, 1.44)	2.4 (1.0,5.6)
	5th quantile	132	29	1	1
Decision about MCH care	Husband/partner	133	5	1	1
	Jointly	573	61	2.8 (1.1, 7.2)	3.6 (1.0,12)*
	Respondents	33	2	1.6 (0.3, 8.7)	1.0 (0.1, 8)
ANC visit	No visit	97	2	1	1
	1–3 visits 4 & more visits	301 339	32 34	5.2 (1.2, 22) 4.9 (1.1, 21)	1.5 (0.2, 11) 0.6 (0.08,4.6)
BPCR^a^ practice	No	619	17	1	1
	Yes	120	51	15.4 (8.6, 27.7)	6.5 (2.3,18)*
Place of previous childbirth	Home-birth	494	5	1	1
	Health facility birth	234	63	26.2 (10, 67)	12 (3.8, 40)*
Previous Childbirth complications	No	708	60	1	1
	Yes	31	8	3.0 (1.3,6.9)	3 (1.0,9)*
Travel distance to the nearest hospital	< 2 h	253	57	9.9 (5,19.3)	3.3 (1.4,7.7)*
	≥ 2 h	486	11	1	1
Emergency transportation access	Easy to access	84	46	16.3 ( 9.3, 28)	8.8 (3.9, 19)*
	Difficult to access	655	22	1	1

*Significant at *p *< 0.05

^a^BPCR- Birth Preparedness and Complication Readiness

^b^Adjusted for confounders which possibly affect the association between the outcome and independent variables

## Discussion

Using mixed methods design, this study tried to explore the barriers to the use of MWHs at Arba Minch Zuria District. Although all health centers in the study area have MWHs, the study revealed that only 8.4% of the participants used the service for their last pregnancy. The women’s economic status, decisions made jointly with male partners (husbands) for obstetric emergency, history of previous institutional childbirth, birth preparedness plan practice, history of previous childbirth complications, < 2hrs travel distance to the nearest HI, and ease of access to transport in case of obstetric emergency were independent factors significantly associated with MWH use.

The level of MWH use in agreement with a previous study in Jimma Zone, Western Ethiopia in 2016, where only 7% of the study participants had ever used MWHs [34]. In rural and hard-to-reach areas, the WHO recommends bringing all pregnant mothers, particularly those who are at risk of childbirth complications, physically close to obstetric facilities. The current study was based entirely on the rural area, with the findings showing that the current use of MHW services is very low, which is also supported by other previous studies [35, 36]. This might be due to the low level of awareness regarding the importance and benefits of using MWH, inadequate referral from health posts where pregnant women get in contact with the health system, poor encouragement of pregnant women to make prior arrangements for MWH services during antenatal care as part of their personal birth preparedness plans, and the perceived poor condition of existing MWHs [37].

The findings from a study conducted in Zambia showed a 31.5% MWH utilization, which is higher than for the current study. This is probably because promotional and awareness-creation activities might have been done, apart from the routine care, as part of the Saving Mothers Giving Life project in the Zambian study. Moreover, one possible reason why a lower MWH use was recorded in the current study might have been because most MWHs are inaccessible due to the mountainous nature of the setting and lack of faster transport options [38].

This study showed that women in the lower wealth quintiles (1st and 2nd) were more likely to use MWH services than those women in the highest (5th) quantile. Similar findings have also been reported by studies conducted in Gurage zone, Central Ethiopia, Tanzania, Zambia and Malawi, all of which highlighted that poorer women were more likely to use MWH services than women in the wealthiest quintile [14, 39–42]. The possible reason might be that staying in MWHs may be the only means the poorer women could access obstetric care and overcome delay in reaching the HIs as a result of exorbitant transportation cost and poor road condition. It is also likely that women from households in the lowest quintile had no choice than to stay in MWHs even though the existing MWHs are substandard. At the same time, the poor quality of MWHs service might have discouraged the richest women from utilizing the existing MWHs.

On the contrary, a cross-sectional study conducted in Jimma Zone, Western Ethiopia reported that women from wealthier households were more likely to be MWH-users than the poorest women [34], notwithstanding that the wealthy women (5th quintile) would likely have the economic means for emergency transport to the hospital once they went into labor. This result suggests that women in the wealthier quintiles can be encouraged to use MWHs as far as the problem related to the quality of service is resolved. Moreover, there is the need to upgrade the condition of the existing MWHs to a level suitable for women from different socioeconomic backgrounds.

The current study also found that women who made decisions jointly with their husbands had higher odds of staying in MWHs compared to those women whose husbands alone took the decisions. This finding is also supported by the qualitative finding that most MWH users were accompanied and financially supported by their husbands. Also, MWH users explained that it would not be possible for a woman to stay in MWHs without a joint decision with the husband, as the woman is expected to leave the house for a longer period; the husband and other adult care-takers would have to take care of the home chores till the mother returns back home. A number of studies indicated that decisions made jointly with husbands/partners were associated with improved maternal health outcomes, including maternal service utilization [43, 44]. This shows that household decision-making to seek obstetric care from the HIs, particularly to stay in MWHs, in collaboration with husbands, could increase the use of MWHs.

Another factor which was statistically associated with use of MWH services was the practice of birth preparedness plan. The finding showed that even though staying at MWH is not a component of personalized birth preparedness plan, women who had prepared for birth and its possible complications were about 6.5 times more likely to use MWH services than those who had not [5]. This is due to the fact that in rural areas like Arba Minch Zuria District, lack of transportation options and poor accessible roads are major hindrances to access to lifesaving obstetric care in case of emergency. As a result, those pregnant women who prepared to give birth at HIs preferred to stay at MWHs until they were due for childbirth. It is also likely that those women who practiced birth preparedness plan received enough counseling from the healthcare providers, which might include the use of MWH services. Hence, the inclusion of MWHs as a component of birth preparedness plan in the rural areas may be necessary.

Because birth complications are unpredictable, pregnant women who are at a risk of complications related to childbirth are encouraged to use MWH services [5]. Previous studies have reported that most intended MWH users had experienced previous childbirth complications [40] or anticipated delivery complications and so needed to be close to HIs [34]. The result of the present study is consistent with those of these previous studies, highlighting that those mothers who had experienced childbirth complications were more likely to use MWH services during the following pregnancy.

This implies that women who have had birth complications may be afraid that they are at a risk of similar events during the following childbirth, so that they decide to be close to the HIs by staying at MWHs. On the contrary, one study carried out in Gurage Zone, Ethiopia reported that pregnancy complications were not statistically associated with the MWH utilization. This might be due to the differences in the study setting, as the current study was entirely based in a rural area, where the study population might not have had alternative means to overcome the second delay but to stay at MWH [45].

Earlier studies have shown a significant association between the use of MWHs and an increase in facility delivery, antenatal and postnatal attendance [16, 46, 47]. This result is somehow related to our finding that those women who had a history of previous health institutional childbirth were more likely to use MWHs than those who used to give birth outside the HIs. Women who have a history of previous health institutional childbirth (usually due to difficulty during labor) are likely to get adequate counseling and advice from skilled birth attendants if they stay at MWHs during their next pregnancies. Lessons the women learned from their past experiences might have informed their decisions to use the MWHs. The role of the health professionals in counseling the women may also be significant in this regard.

MWHs were established to bridge the geographic gap in accessing obstetric care in remote areas [5]. However, our findings showed that those women residing in areas within 2 h of walking distance to the nearest HI were more likely to use MWH service than those women whose homes were more than 2 h of walking distance from the nearest HI. Similarly, those women who had easy access to transport in case of obstetric emergency were more likely to use MWH service than those women who had difficulty obtaining means of transport. A study conducted in rural Timor-Leste in 2007 also found a comparable result that women who lived within 5 km of the health centers were the group most likely to use MWHs [48]. The potential barriers to the utilization of MWHs, including long distances and the unavailability of means of transport to and from the MWHs, could be the reason why women in remote areas are not motivated to come to MWHs. Moreover, women in remote areas may not have adequate information about the benefits of MWHs and the adverse consequences of home delivery [38].

This conveys a message to the concerned bodies to act urgently as women from remote and hard-to-reach areas, who are the supposed candidates of the MWHs, do not make use of the existing homes probably because of lack of awareness regarding MWHs, difficulty in accessing the homes because of geographical barriers, or total absence of contact with HIs. The results of the current study might be an indication that for improved MWH use, good infrastructure and awareness-creation campaigns are needed to attract women who reside in remote areas [51]. This finding, however, does not corroborate the results of previous studies conducted in Zambia [49], Gurage Zone, Ethiopia [45], and Zimbabwe [50], which found that women who stayed in MWHs were more likely to live further away from the nearest HIs.

This study has two major limitations. First, since the study was cross-sectional, it cannot be proven beyond doubt that the identified factors are causal. Secondly, the women’s self-reported travel time estimates might not have accurately reflected the physical inaccessibility of the MWHs. Although we have tried to encourage participants to actively engage in the FGDs, many of them either remained silent or answered a reply sign to indicate that they share the answer that was given beforehand. One of the reasons could have been their literacy level, being from rural and shyness. Except for these limitations, the study used both quantitative and qualitative methods of data collection, which allowed the exploration of factors that a single method would have not been able to capture adequately and provided up-to-date evidence on the current level of MWH use and its associated factors in the study area.

## Conclusions

A low proportion of women use MWHs in the study area. The socioeconomic status of women, joint decision making involving both the women and their partners, the degree of preparation for birth and possible complications, history of previous obstetric complication, history of previous health institutional childbirth, residence in areas within 2 h of walking distance to the nearest HI, and ease of access to transport in case of obstetric emergency were the independent positive factors significantly associated with the use of MWHs.

Strategies to improve MWH utilization, including health promotion activities, are very crucial to increasing awareness about the importance of MWHs in the health system, thus enabling women to recognize danger signs during pregnancy and encouraging them to take an active role in household decision-making.

Efforts should be made to incorporate MWHs as part of personalized birth preparedness and complication readiness plans for women living very far away from HIs, and to upgrade the existing MWH services so that they are suited to women from all socioeconomic status. As the attitude of healthcare providers has implications for the initial and continued use of MHWs by pregnant women, taking advantage of all opportunities to address the women, especially when they visit the HIs, and counsel them on the importance of MWHs in client-oriented manner are highly recommended. Further researches assessing MWH users experience to better understand the challenges in MWHs use by employing qualitative methods were recommended to bring further evidence in the efforts to end preventable maternal death.

## Supplementary Information

**Additional file 1.** Survey Questionnaire. Survey questionnaire we used to collect information from the study participants.

**Additional file 2.** Focus Group Discussion Guides. The FGD guide we was used to guide the discussions with MWHs users and non-users.

## Data Availability

All data generated during this study are included in this published article and its supplementary information files. The minimum data set is however available from the authors upon reasonable request and with the permission of Arba Minch University.

## Abbreviations

MWH: Maternity waiting home
His: Health institutions
ODK: Open Data Kit
WHO: World Health Organization

## References

[1] WHO, UNICEF, UNFPA, And WBG, D. UNP (2019). Trends in maternal mortality 2000 to 2017.

[2] Say L, Chou D, Barbour K, Barreix M, Cecatti JG, Costa ML (2018). Maternal morbidity: Time for reflection, recognition, and action. Int J Gynecol Obstet..

[3] Lozano R, Wang H, Foreman KJ, Rajaratnam JK, Naghavi M, Marcus JR (2011). Progress towards millennium development goals 4 and 5 on maternal and child mortality: an updated systematic analysis. Lancet.

[4] Central Statistical Agency ICF (2016). A Ethiopia demographic and health survey 2016.

[5] World Health Organization (2015). WHO recommendations on health promotion interventions for maternal and newborn health 2015.

[6] World Health Organization (2017). WHO recommendations on maternal health.

[7] Solnes Miltenburg A, Roggeveen Y, Roosmalen J, Smith H (2017). Factors influencing implementation of interventions to promote birth preparedness and complication readiness. BMC Pregnancy Childbirth.

[8] Thaddeus S, Maine D (1994). Too far to walk: maternal mortality in context. Soc Sci Med.

[9] Geleto A, Chojenta C, Mussa A, Loxton D (2018). Barriers to access and utilization of emergency obstetric care at health facilities in sub-Saharan Africa-a systematic review protocol. Syst Rev..

[10] World Health Organization (1991). Esenstial elements of obstetric care at first referral level.

[11] World Health Organization (1996). Maternity waiting homes: a review of experiences.

[12] Poovan P, Kifle F, Kwast BE (1990). A maternity waiting home reduces obstetric catastrophes. World Health Forum.

[13] Gaym A, Pearson L, Soe KWW (2012). Maternity waiting homes in Ethiopia–three decades experience. Ethiop Med J.

[14] Braat F, Vermeiden T, Getnet G, Schiffer R, van den Akker T, Stekelenburg J (2018). Comparison of pregnancy outcomes between maternity waiting home users and non-users at hospitals with and without a maternity waiting home: retrospective cohort study. Int Health.

[15] Kelly J, Kohls E, Poovan P, Schiffer R, Redito A, Winter H (2010). The role of a maternity waiting area (MWA) in reducing maternal mortality and stillbirths in high-risk women in rural Ethiopia. BJOG An Int J Obstet Gynaecol.

[16] Lori JR, Perosky J, Munro-Kramer ML, Veliz P, Musonda G, Kaunda J (2019). Maternity waiting homes as part of a comprehensive approach to maternal and newborn care: a cross-sectional survey. BMC Pregnancy Childbirth..

[17] Scott NA, Henry EG, Kaiser JL, Mataka K, Rockers PC, Fong RM (2018). Factors affecting home delivery among women living in remote areas of rural Zambia: a cross-sectional, mixed-methods analysis. Int J Womens Health.

[18] Kurji J, Kulkarni MA, Gebretsadik LA, Wordofa MA, Morankar S, Bedru KH (2019). Effectiveness of upgraded maternity waiting homes and local leader training in improving institutional births among women in the Jimma zone, Ethiopia: Study protocol for a cluster-randomized controlled trial. Trials..

[19] Kyomuhendo GB (2003). Low use of rural maternity services in Uganda: Impact of women’s status, traditional beliefs and limited resources. Reprod Health Matters.

[20] Van Lonkhuijzen L, Stekelenburg J, Van Roosmalen J (2009). Maternity waiting facilities for improving maternal and neonatal outcome in low-resource countries. Cochrane Database Syst Rev..

[21] Sialubanje C, Massar K, van der Pijl MSG, Kirch EM, Hamer DH, Ruiter RAC (2015). Improving access to skilled facility-based delivery services: Women’s beliefs on facilitators and barriers to the utilisation of maternity waiting homes in rural Zambia. Reprod Health.

[22] Girma M, Yaya Y, Gebrehanna E, Berhane Y, Lindtjørn B (2013). Lifesaving emergency obstetric services are inadequate in south-west Ethiopia: A formidable challenge to reducing maternal mortality in Ethiopia. BMC Health Serv Res..

[23] Vermeiden T, Braat F, Medhin G, Gaym A, Den Van Akker T, Stekelenburg J (2019). Factors associated with intended Maternity Waiting Home use in Southern Ethiopia. BMJ Open..

[24] Mramba L, Nassir FA, Ondieki C, Kimanga D (2010). Reasons for low utilization of a maternity waiting home in rural Kenya. Int J Gynecol Obstet.

[25] Haile D, Kondale M, Andarge E, Tunje A, Fikadu T, Boti N (2019). Level of completion along continuum of care for maternal and child health services and factors associated with it among women in Arba Minch Zuria Woreda, Gamo Zone, Southern Ethiopia: a community based cross-sectional study. PLoS ONE.

[26] Gurara M, Muyldermans K, Jacquemyn Y, Van Geertruyden JP, Draulans V (2019). Traditional birth attendants’ roles and homebirth choices in Ethiopia: a qualitative study. Women Birth..

[27] Andarge E, Nigussie A, Wondafrash M (2017). Factors associated with birth preparedness and complication readiness in Southern Ethiopia : a community based cross-sectional study. BMC Pregnancy Childbirth.

[28] Arifin WN (2013). Introduction to sample size calculation. Educ Med J..

[29] Kurji J, Gebretsadik LA, Wordofa MA, Sudhakar M, Asefa Y, Kiros G (2019). Factors associated with maternity waiting home use among women in Jimma Zone, Ethiopia: a multilevel cross-sectional analysis. BMJ Open..

[30] Central Statistical Agency (CSA) [Ethiopia] and ICF (2016). Ethiopian Demographic and Health Survey 2016.

[31] Debelew GT, Afework F, Yalew AW (2014). Factors affecting birth preparedness and complication readiness in Jimma Zone, Southwest Ethiopia: a multilevel analysis. Pan Afr Med J.

[32] Del Barco RC (2004). Monitoring birth preparedness and complication readiness. Tools and indicators for maternal and newborn health.

[33] Kaso M, Addisse M (2014). Birth preparedness and complication readiness in Robe Woreda, Arsi Zone, Oromia Region, Central Ethiopia: a cross-sectional study. Reprod Health.

[34] Kurji J, Gebretsadik LA, Wordofa MA, Sudhakar M, Asefa Y, Kiros G (2019). Factors associated with maternity waiting home use among women in Jimma Zone, Ethiopia: a multilevel cross-sectional analysis. BMJ Open.

[35] Lee ACC, Lawn JE, Cousens S, Kumar V, Osrin D, Bhutta ZA (2009). Linking families and facilities for care at birth: What works to avert intrapartum-related deaths?. Int J Gynecol Obstet..

[36] Singh K, Speizer I, Kim ET, Lemani C, Phoya A (2017). Reaching vulnerable women through maternity waiting homes in Malawi. Int J Gynaecol Obstet.

[37] Lori JR, Boyd CJ, Munro-Kramer ML, Veliz PT, Henry EG, Kaiser J (2018). Characteristics of maternity waiting homes and the women who use them: findings from a baseline cross-sectional household survey among SMGL-supported districts in Zambia. PLoS ONE.

[38] Kebede KM, Mihrete KM (2020). Factors influencing women’s access to the maternity waiting home in rural Southwest Ethiopia: a qualitative exploration. BMC Pregnancy Childbirth.

[39] Sialubanje C, Massar K, Hamer DH, Ruiter RAC (2017). Personal and environmental factors associated with the utilisation of maternity waiting homes in rural Zambia. BMC Pregnancy Childbirth..

[40] Vermeiden T, Braat F, Medhin G, Gaym A, van den Akker T, Stekelenburg J (2018). Factors associated with intended use of a maternity waiting home in Southern Ethiopia: a community-based cross-sectional study. BMC Pregnancy Childbirth.

[41] Fogliati P, Straneo M, Mangi S, Azzimonti G, Kisika F, Putoto G (2017). A new use for an old tool: Maternity waiting homes to improve equity in rural childbirth care. Results from a cross-sectional hospital and community survey in Tanzania. Health Policy Plan..

[42] Singh K, Speizer IS, Kim ET, Lemani C, Tang JH, Phoya A (2018). Evaluation of a maternity waiting home and community education program in two districts of Malawi. BMC Pregnancy Childbirth.

[43] Eshete A, Adissu Y (2017). Women’s joint decision on contraceptive use in gedeo zone, southern ethiopia: a community based comparative cross-sectional study. Int J Family Med.

[44] Ghose B, Feng D, Tang S, Yaya S, He Z, Udenigwe O (2017). Women’s decision-making autonomy and utilisation of maternal healthcare services: Results from the Bangladesh Demographic and Health Survey. BMJ Open.

[45] Getachew B, Liabsuetrakul T, Gebrehiwot Y (2020). Association of maternity waiting home utilization with women’s perceived geographic barriers and delivery complications in Ethiopia. Int J Health Plann Manage.

[46] Henry EG, Semrau K, Hamer DH, Vian T, Nambao M, Mataka K (2017). The influence of quality maternity waiting homes on utilization of facilities for delivery in rural Zambia. Reprod Health..

[47] Lori JR, Munro ML, Rominski S, Williams G, Dahn BT, Boyd CJ (2013). Maternity waiting homes and traditional midwives in rural Liberia. Int J Gynecol Obstet.

[48] Wild K, Barclay L, Kelly P, Martins N (2012). The tyranny of distance: Maternity waiting homes and access to birthing facilities in rural Timor-Leste. Bull World Health Organ.

[49] Van Lonkhuijzen L, Stegeman M, Nyirongo R, Van Roosmalen J (2003). Use of maternity waiting home in rural Zambia. Afr J Reprod Health.

[50] Chandramohan D, Cutts F, Millard P (1995). The effect of stay in a maternity waiting home on perinatal mortality in rural Zimbabwe. J Trop Med Hyg.

[51] Bergen N, Abebe L, Asfaw S, Kiros G, Kulkarni MA, Mamo A (2019). Maternity waiting areas–serving all women? Barriers and enablers of an equity-oriented maternal health intervention in Jimma Zone, Ethiopia. Glob Public Health.

