# Supplementary material for: Maternity waiting homes as component of birth preparedness and complication readiness for rural women in hard-to-reach areas in Ethiopia
Source: Reprod Health. 2021 Feb 2;18:27. doi: 10.1186/s12978-021-01086-y (PMC7856798; doi:10.1186/s12978-021-01086-y)
Supplement: Supplementary file 1 — Additional file 1. Survey Questionnaire. Survey questionnaire we used to collect information from the study participants. [file 12978_2021_1086_MOESM1_ESM.docx]

# Additional file 1 English version questionnaire

**Arba Minch University College of Medicine and Health Sciences**

Questionnaire to assess the level of utilization and barriers to use maternity waiting homes among women who have been given birth two years preceding the study in Arba Minch Zuria districts, Gamo Gofa Zone, Southern Ethiopia

Hello! My name is _______________I am one of the data collectors for the research team investigating MWHs in AMU HDSS site. The purpose of this questionnaire is to gather information on level of utilization and barriers to use maternity waiting homes among women who have been given birth two years preceding the study in Arba Minch Zuria districts, Gamo Gofa Zone, Southern Ethiopia

I have identified you as a study participant hoping that you would be willing to help me by providing with some information. I have several questions which I would like to ask you, if you have the time and are willing. All information you provide will be kept confidential. I will not include any identifiers, such as your name or exact address. Only honest answers would contribute to improvement of health planning. Your role in the success of the research is important and I appreciate your contribution to the research. Would this be okay with you?

I understood about the advantage of the research and the roles I will have in the research. Have you agreed to participate in the research?

Yes No

Date _________________________

Sig. __________________________

# Part I Questions related to Socio-demographic characteristics of the respondents

| **S.No** | **Question** | **Response** | **Remarks** |
| --- | --- | --- | --- |
| 101 | Age in completed years | _______________ years |  |
| 102 | To which ethnic group do you belong? | 1. Gamo 2. Wolayta 3. Zeyse 4. Amhara 5. Other (Specify) ____________ |  |
| 103 | What is your religion? | 1. Orthodox 2. Protestant 3. Muslim 4. Traditional 5. Other (Specify) ______________ |  |
| 104 | What is the highest grade you completed? | 1. Illiterate 2. Read and write 3. Elementary school (grade 1 -4) 4. Secondary school(Grade 5-8) 5. High school/prep.(grade 9 -12) 6. Above grade 12 |  |
| 105 | What is your occupation? | 1. Housewife 2. Government employee 3. Private business 4. Daily laborer 5. Farmer 6. Merchant 7. Other Specify)______________ |  |
| 106 | What is your marital status? | 1. Married 2. Single 3. Divorced 4. Widowed 5. Separated |  |
| 107 | Your husband‘s/ partner’s age in completed years | ____years |  |
| 108 | What is the educational level of your husband? | 1. Illiterate 2. Read and write 3. Elementary school (grade 1 -4) 4. Secondary school(Grade 5-8) 5. High school/prep.(grade 9 -12) 6. Above grade 12 |  |
| 109 | What is the occupation of your husband? | 1. Merchant 2. Government employee 3. Private business 4. Daily laborer 5. Farmer 6. Unemployed 7. Other (specify) |  |
| 110 | Family size | ________ |  |
| 111 | Who usually decides how the family earnings are spent? | 1. Respondent 2. Husband/partner 3. Jointly |  |

# Part II: Questions related to Reproductive and Obstetric Characteristics of the respondents

| **S.No** | | **Question** | **Response** | **Remarks** |
| --- | --- | --- | --- | --- |
| **Part II a. last pregnancy** | | | | |
| 201 | What was your age at your first pregnancy? | | 1. Age in years____ 2. I don’t remember |  |
| 202 | How many times did you get pregnant?(Gravidity) | | _______________ times |  |
| 203 | How many times have you given birth to a child in the past five years? | | _______________ times |  |
| 204 | Number of still born in the past five years? | | _______________ times |  |
| 205 | Number of abortion in the past five years? | | _______________ times |  |
| 206 | Have you ever given birth to a boy or girl who was born alive but died within the first week in the past five years? | | 1. Yes 2. No |  |
| 207 | If yes to Q207, where was the neonate born? | | 1. Home 2. Health institution 3. Other(specify) ______ |  |
| 208 | How old is your last child? | | ____completed years |  |

# Part: III Questions related to maternal health service utilization

| **S.No** | **Question** | **Response** | **Remarks** |
| --- | --- | --- | --- |
| 302 | Have you ever used anything or tried in any way to delay or avoid getting pregnant? | 1. Used in the past 2. Never used |  |
| 303 | During the pregnancy with your last child pregnancy, do you have antenatal care follow up? | 1. Yes 2. No | If yes, Skip to Q305 |
| 304 | If Q303 is no, can you tell us the reasons? (Multiple answer is possible) | 1. No/ little Knowledge about ANC 2. No health problem encountered 3. Health institution is too far from my home 4. Expense to ANC is unaffordable. 5. Long waiting time 6. Poor handling by health care providers 7. Lack of transportation 8. Lack of time 9. Other (specify)_______ |  |
| 305 | How many times did you receive antenatal care during you’re your last pregnancy? | __________times (ANC Visits) |  |
| 306 | At what gestational age did you start pregnancy checkup of the last pregnancy? | ______________GA/week |  |
| 309 | Where you told the signs of pregnancy complications during you ANC visits? | 1. Yes 2. No |  |
| 310 | Can you tell me any signs of pregnancy complications? | 1. Vaginal bleeding 2. Vaginal flush of fluid 3. Severe headache, 4. Blurred vision, 5. Fever 6. Abdominal pain/pre- term contractions, 7. Decreased fetal movement, 8. Edema/ body swelling 9. Other, specify |  |
| 311 | Where you advised about birth preparedness? | 1. Yes 2. No |  |
| 312 | During your last pregnancies, did you plan in advance where you wanted to give birth? | 1. Yes 2. No |  |
| 313 | Where did you plan to give birth? | 1. Health facility 2. Home |  |
| 314 | Did you identify a trained birth attendant? | 1. Yes 2. No |  |
| 315 | Did you identify a health facility (for an obstetric emergency)? | 1. Yes 2. No |  |
| 316 | Did you identify a mode of transport to the facility (for obstetric emergency)? | 1. Yes 2. No |  |
| 317 | Did you save money for the delivery and/or an emergency? | 1. Yes 2. No |  |
| 318 | Did you identify a blood donor? | 1. Yes 2. No |  |
| 319 | Where do believe that you run the greatest risk during a delivery, at home or in a health facility? | 1. Home 2. Health facility |  |
| 320 | Where do you believe that your baby runs the greatest risk during a delivery, at home or in a health facility? | 1. Home 2. Health facility |  |
| 321 | What is the travel time from your household to the nearest Health Post? | 1. Minutes 2. Hours 3. No HP |  |
| 322 | What is the travel time from your household to the nearest Health Center? | 1. Minutes 2. Hours 3. No HC |  |
| 323 | What is the travel time from your household to the nearest hospital? | 1. Minutes 2. Hours |  |
| 324 | Once labor has started at home, how easy or difficult is it to find transport? | 1. Very easy 2. Easy 3. Difficult 4. Very difficult |  |
| 325 | In case of an emergency during home delivery, what mode of transport is used for the majority of the journey to the nearest hospital? | 1. Walking / Carried 2. Horse 3. Public transport 4. Private transport 5. Ambulance |  |
| 326 | Where did you give birth to your last child? | 1. At health institution (health center or hospital) 2. At home 3. On the way to health institutions 4. On farm/working area 5. Other (specify)________ |  |
| 327 | If Not at health institutions, Why? | 1. Because the labour was very fast 2. I did not had transportation money 3. I prefer home delivery 4. The ambulance came late 5. Cost too much 6. Facility (often) not open / possibility of onward referrals 7. Don’t trust facility 8. Poor quality of service 9. No female provider at facility 10. Husband/family did not allow 11. No family members allowed 12. No privacy 13. No cultural rituals 14. No complications 15. Fear of an operation 16. Other (Specify) ___________ |  |
| 311 | Who assisted you in the last delivery? | Health professionalTraditional birth attendantFamily memberNo one (self)Other (Specify) |  |
| 312 | Why did you deliver in a health institution? | 1. Comfortable 2. Privacy 3. Complications in previous pregnancies/deliveries 4. Complication during labor 5. Husband/ family decided 6. Low cost 7. Other specify |  |
| 328 | If your last delivery was at the health institutions, which was the mode of delivery for your last child? | 1. Spontaneous vaginal delivery 2. Instrumental delivery 3. Caesarian section 4. Other (specify) ___ |  |
| 329 | Have you experienced any complications during your last delivery? | 1. Yes 2. No |  |
| 330 | If yes, which complications? Multiple responses are allowed | 1. Hemorrhage 2. Prolonged labour 3. Obstructed labour 4. Hypertensive disorder 5. Puerperal infection 6. Fetal distress 7. Intrauterine Fetal Death 8. other specify |  |
| 331 | Did you seek medical advice or treatment for these complications? | 1. Yes 2. No | If place of delivery Not at health institutions, skip to 334 |
| 332 | Where did you seek medical advice or treatment for these complications? | 1. TBA 2. Health facility 3. Other, namely |  |
| 333 | If no to Q316, Why didn’t you seek medical advice or treatment for these complications? | Cost too much  Facility (often) not open / possibility of onward referrals  Too far/no transportation  Don’t trust facility  Poor quality of service  No female provider at facility  Husband/family did not allow  No family members allowed  No privacy  Not necessary  Not customary  Other, namely |  |
| 334 | Did you get medical checkup after your last delivery within 42days? | 1. Yes 2. No 3. I don‘t remember |  |
| 335 | If yes to Q334 when did you get it for the first time after delivery | After ______ hrs of delivery  After ______ days of delivery  After ______ weeks of delivery |  |
| 336 | Where did you get the checkup for the first time? | 1. Home 2. Hospital 3. HC 4. Health post 5. Others specify___ |  |

# Part IV: Awareness, experience and attitude towards Maternity Waiting Homes

|  | | | |
| --- | --- | --- | --- |
| 401 | Have you ever heard of Maternity Waiting Homes? | 1. Yes 2. No |  |
| 402 | If Yes, from where did you get the information? | 1. HEWs 2. HC staff 3. HDA leaders 4. Community leaders 5. Other _________ |  |
| 403 | Do you know that the availability of MWH service in the nearest HC? | 1. Yes 2. No |  |
| 404 | Did you ever stay at Maternity Waiting Homes? | 1. Yes 2. No | If NO, skip to 501. |
| 405 | What were the reasons for your admission to the MWH? | 1. Previous caesarean section 2. Previous obstetric fistula repair 3. Multiple pregnancy 4. Grand multi-parity 5. Previous stillbirth/neonatal loss 6. Mal-presentations 7. Breech presentation 8. Antepartum hemorrhage 9. Anemia 10. Preeclampsia/medical problems 11. Living far away from a hospital with emergency obstetrics care 12. Other, specify: |  |
| 406 | From where were you referred to the MWH? | 1. HEWs 2. HP 3. HC 4. Hospital 5. Other, specify |  |
| 407 | How long did you stay in the MWH? | Length of stay_______________ |  |
| 408 | Who made the decision to stay at the MWH? | 1. Respondent 2. Husband/partner 3. Jointly 4. Other, specify |  |
| 409 | Who was your attendant during your stay at the MWH? | 1. Husband/partner 2. Other family member 3. Other, specify |  |
| 410 | Who provided financial support during your stay at the MWH? (Transport, food, medications) | 1. Respondent 2. Husband 3. Other family member 4. Other, specify |  |
| 411 | Who provided social support during your stay at the MWH? (Taking care of children, household chores and/or other work, emotional support) | 1. Husband 2. Other family member 3. Other, specify |  |
| 412 | What do you think the advantages of staying at MWH?  Multiple answers are possible. | 1. Closeness to emergency obstetrics 2. care 3. Saving life of mother 4. Saving life of baby 5. Calmness, rest before delivery 6. Other, specify   99. Don’t know |  |
| 413 | For your current/next pregnancy, how likely is it that you will stay at a MWH the last 2-4 weeks prior to your delivery? | 1. Very likely 2. Likely 3. Somewhat unlikely 4. Very unlikely |  |
| 414 | Barriers to maternity waiting homes. Multiple answers are possible. | 1. Transport to and from the MWH 2. Food while staying at MWH 3. Bringing own cooking utensils to MWH 4. Stay at MWH 2–4 weeks before delivery 5. Bringing an attendant for at least 6. 2 weeks before delivery date 7. Children are taken care of by other family members /community 8. Household chores are taken care of by family / community 9. Being away from your work (other than household chores) 10. Attendant being away from other work / obligations 11. Bringing a child to MWH 12. Visits from family members 13. Other challenges, specify |  |
| 415 | Perceived quality MWH facilities | 1. Good 2. Satisfactory 3. Reasonable 4. Poor |  |
| 416 | Quality MWH hygiene | 1. Good 2. Satisfactory 3. Reasonable 4. Poor |  |
| 417 | Perceived quality MWH privacy | 1. Good 2. Satisfactory 3. Reasonable 4. Poor |  |
| 418 | Perceived quality MWH support women | 1. Good 2. Satisfactory 3. Reasonable 4. Poor |  |
| 419 | Perceived quality MWH space attendants | 1. Good 2. Satisfactory 3. Reasonable 4. Poor |  |
| 420 | Would you recommend the MWH to other pregnant women? | Yes  No |  |
| 421 | MWH are very important for pregnant women | 1. Strongly disagree 2. Disagree 3. Neutral Agree 4. Strongly agree |  |
| 422 | MWHs should be available in every HC | 1. Strongly disagree 2. Disagree 3. Neutral Agree 4. Strongly agree |  |
| 423 | Maternity waiting home is against our tradition/culture | 1. Strongly disagree 2. Disagree 3. Neutral Agree 4. Strongly agree |  |
| 424 | MWHs affect the life of the rest children negatively | 1. Strongly disagree 2. Disagree 3. Neutral Agree 4. Strongly agree |  |
| 425 | MWHs is not good because it separates mothers from other children | 1. Strongly disagree 2. Disagree 3. Neutral Agree 4. Strongly agree |  |
| 426 | MWHs are not god because it forces women of different conduct to live together | 1. Strongly disagree 2. Disagree 3. Neutral Agree 4. Strongly agree |  |
| 427 | MWHs are ideal place to share information about pregnancy and childbirth | 1. Strongly disagree 2. Disagree 3. Neutral Agree 4. Strongly agree |  |
| 428 | I do not prefer MWHs because the HC staffs humiliate the mothers because they lack urban life style | 1. Strongly disagree 2. Disagree 3. Neutral Agree 4. Strongly agree |  |
| 429 | I do not prefer MWHs because of fear of acquiring infections from other mothers | 1. Strongly disagree 2. Disagree 3. Neutral Agree 4. Strongly agree |  |
| 430 | MWHs are totally not important at all | 1. Strongly disagree 2. Disagree 3. Neutral Agree 4. Strongly agree |  |

# Part V. Wealth Index Questions

|  | | | |
| --- | --- | --- | --- |
| S/N | Question | Alternative | Skip |
|  | What is the main source of drinking water for members of your household? | 1. Piped Water    1. Piped into Dwelling    2. Piped to Yard/Plot    3. Piped to Neighbor    4. Public Tap/Standpipe 2. Tube Well or Borehole 3. Dug Well    1. Protected Well    2. Unprotected Well 4. Water from Spring    1. Protected Spring    2. Unprotected Spring 5. Rainwater 6. Tanker Truck (Boti) 7. Cart with Small Tank 8. Surface Water (River/Dam/Lake/ Pond/Stream/Canal/Irrigation Channel) 9. Bottled Water 10. Other (Specify ) | 106  106  106 |
|  | What is the main source of water used by your household for other purposes such as cooking and hand washing? | 1. Piped Water    1. Piped into Dwelling    2. Piped to Yard/Plot    3. Piped to Neighbor    4. Public Tap/Standpipe 2. Tube Well or Borehole 3. Dug Well    1. Protected Well    2. Unprotected Well 4. Water from Spring    1. Protected Spring    2. Unprotected Spring 5. Rainwater 6. Tanker Truck (Boti) 7. Cart with Small Tank 8. Surface Water (River/Dam/Lake/ Pond/Stream/Canal/Irrigation Channel) 9. Bottled Water 10. Other (Specify ) |  |
|  | Where is that water source located? | 1. IN OWN DWELLING 2. IN OWN YARD/PLOT 3. ELSEWHERE |  |
|  | How long does it take to go there, get water, and come back? | MINUTES___________  DON'T KNOW |  |
|  | Who usually goes to this source to fetch the water for your household? | 1. ADULT WOMAN 2. ADULT MAN 3. FEMALE CHILD    1. UNDER 15 YEARS OLD 4. MALE CHILD    1. UNDER 15 YEARS OLD 5. OTHER (specify) |  |
|  | In the past two weeks, was the water from this source not available for at least one full day? | 1. Yes 2. No 3. Don’t know |  |
|  | Do you do anything to the water to make it safer to drink? | 1. Yes 2. No 3. Don’t know |  |
|  | What do you usually do to make the water safer to drink?  Anything else?  RECORD ALL MENTIONED | 1. Boil 2. Add Bleach/Chlorine 3. Strain Through A Cloth 4. Use Water Filter (Ceramic/Sand/Composite/Etc) 5. Solar Disinfection 6. Let It Stand And Settle 7. Other (Specify) 8. Don't Know |  |
|  | What kind of toilet facility do members of your household usually use?  IF NOT POSSIBLE TO DETERMINE, ASK PERMISSION TO OBSERVE THE FACILITY. | 1. Flush or Pour Flush Toilet    1. Flush to Piped Sewer System    2. Flush to Septic Tank    3. Flush to Pit Latrine    4. Flush to Somewhere Else    5. Flush, Don't Know Where 2. Pit Latrine    1. Ventilated Improved Pit Latrine    2. Pit Latrine with Slab    3. Pit Latrine Without Slab/Open Pit 3. Composting Toilet 4. Bucket Toilet 5. Hanging Toilet/Hanging Latrine 6. No Facility/Bush/Field 7. Other (Specify) |  |
|  | Do you share this toilet facility with other households? | 1. Yes 2. No |  |
|  | Including your own household, how many households use this toilet facility? | NO. OF HOUSEHOLDS |  |
|  | Where is this toilet facility located? | 1. IN OWN DWELLING 2. IN OWN YARD/PLOT 3. ELSEWHERE |  |
|  | What type of fuel does your household mainly use for cooking? | 1. Electricity 2. Lpg 3. Natural Gas 4. Biogas 5. Kerosene 6. Charcoal 7. Wood 8. Straw/Shrubs/Grass 9. Agricultural Crop 10. Animal Dung 11. No Food Cooked In Household 12. Other (Specify) |  |
|  | Is the cooking usually done in the house, in a separate building, or outdoors? | 1. In the House 2. In A Separate Building 3. Outdoors 4. Other (Specify) |  |
|  | Do you have a separate room which is used as a kitchen? | 1. Yes 2. No |  |
|  | How many rooms in this household are used for sleeping? | ROOMS |  |
|  | Does this household own any livestock, herds, other farm animals, or poultry? | 1. Yes 2. No |  |
|  | How many of the following animals does this household own? | 1. Milk cows, oxen or bulls? _______ 2. Other cattle? _______ 3. Horses, donkeys, or mules? _______ 4. Camels _______ 5. Goats? _______ 6. Sheep? _______ 7. Chickens or other poultry? _______ 8. Beehives? _______ |  |
|  | Does any member of this household own any agricultural land? | 1. Yes 2. No |  |
|  | How many hectares of agricultural land do members of this household own? | HECTARES________ |  |
|  | Does your household have: | 1. Ownership of owned living house Y/N 2. Plough plow Y/N 3. Axe Y/N 4. Hoe Y/N 5. Shovel Y/N 6. Sickle Y/N 7. Modern beehive Y/N 8. Traditional beehive Y/N |  |
|  | Does your household have: | 1. A table? Y/N 2. A chair? Y/N 3. A bed with cotton/sponge/spring mattress? Y/N 4. A refrigerator? Y/N 5. A non-mobile telephone? Y/N 6. A computer? Y/N 7. A television? Y/N 8. A radio? Y/N 9. Electricity? Y/N 10. An electric mitad? Y/N 11. A kerosene lamp/pressure lamp? Y/N |  |
|  | Does any member of this household own | 1. A boat with a motor? Y/N 2. An animal-drawn cart? Y/N 3. A car or truck? Y/N 4. A watch? Y/N 5. A mobile phone? Y/N 6. A bajaj? Y/N 7. A motorcycle or motor scooter? Y/N 8. A bicycle? Y/N |  |
|  | Does any member of this household own |  |  |
|  | Does any member of this household have a bank account? | 1. Yeas 2. No |  |
|  | How often does anyone smoke inside your house? Would you say daily, weekly, monthly, less often than once a month, or never? | 1. Daily 2. Weekly 3. Monthly 4. Less Often Than Once A Month 5. Never |  |

Thank you for taking the time to talk to us!!
