# Supplementary material for: Maternity waiting homes as component of birth preparedness and complication readiness for rural women in hard-to-reach areas in Ethiopia
Source: Reprod Health. 2021 Feb 2;18:27. doi: 10.1186/s12978-021-01086-y (PMC7856798; doi:10.1186/s12978-021-01086-y)
Supplement: Supplementary file 2 — Additional file 2. Focus Group Discussion Guides. The FGD guide we was used to guide the discussions with MWHs users and non-users. [file 12978_2021_1086_MOESM2_ESM.docx]

# Additional file 2 Focus Group Discussion (FGD) Questions

Selection criteria

Women who have at least one child and lived in the community for at least six months and used MWHs (Group 1) and did not use MWHs (Group 2)

Consent to Participate in Focus Group Discussion

Hello! May name is ________________ I am one of the research teams from Arba Minch University who are conducting research on Maternity Waiting Homes in Arba Minch HDSS: Level of Utilization and its barriers and one of data collection means is focus group discussion.

You have been asked to participate in a focus group organized by research team from Arba Minch University. The purpose of the group discussion is to explore opinions, feeling, and personal experiences about Maternity Waiting Homes. The information learned in the focus groups will be used as an input for the improvement of maternal and child health of this community.

You can choose whether or not to participate in the focus group and stop at any time. Although the focus group will be tape recorded, your responses will remain anonymous and no names will be mentioned in the report. There is no right or wrong answers to the focus group questions. We want to hear many different viewpoints and would like to hear from everyone. We hope you can be honest even when your responses may not be in agreement with the rest of the group. In respect for each other, we ask that only one individual speak at a time in the group and that responses made by all participants be kept confidential. I understand this information and agree to participate fully under the conditions stated above:

Signed:____________________________________________ Date:___________________

Socio-demographic characteristics of the FGD participants

1. Sex
   1. Male
   2. Female

| P1 | P2 | P3 | P4 | P5 | P6 | P7 | P8 | P9 | P10 |
| --- | --- | --- | --- | --- | --- | --- | --- | --- | --- |
|  |  |  |  |  |  |  |  |  |  |

1. Age _____ Years

| P1 | P2 | P3 | P4 | P5 | P6 | P7 | P8 | P9 | P10 |
| --- | --- | --- | --- | --- | --- | --- | --- | --- | --- |
|  |  |  |  |  |  |  |  |  |  |

1. Occupation
   1. Famer
   2. House wife
   3. Government employee specify profession _______________
   4. Private business
   5. Other (Specify)

| P1 | P2 | P3 | P4 | P5 | P6 | P7 | P8 | P9 | P10 |
| --- | --- | --- | --- | --- | --- | --- | --- | --- | --- |
|  |  |  |  |  |  |  |  |  |  |

1. Educational status
   1. Illiterate
   2. Read and write
   3. Elementary school (grade 1 -4)
   4. Secondary school(Grade 5-8)
   5. High school/prep.(grade 9 -12)
   6. Above grade 12

| P1 | P2 | P3 | P4 | P5 | P6 | P7 | P8 | P9 | P10 |
| --- | --- | --- | --- | --- | --- | --- | --- | --- | --- |
|  |  |  |  |  |  |  |  |  |  |

1. Parity ( for female participants oly) _________

| P1 | P2 | P3 | P4 | P5 | P6 | P7 | P8 | P9 | P10 |
| --- | --- | --- | --- | --- | --- | --- | --- | --- | --- |
|  |  |  |  |  |  |  |  |  |  |

1. Religion
   1. Orthodox
   2. Protestant
   3. Muslim
   4. Other (Specify)

| P1 | P2 | P3 | P4 | P5 | P6 | P7 | P8 | P9 | P10 |
| --- | --- | --- | --- | --- | --- | --- | --- | --- | --- |
|  |  |  |  |  |  |  |  |  |  |

1. Ethnic group____

| P1 | P2 | P3 | P4 | P5 | P6 | P7 | P8 | P9 | P10 |
| --- | --- | --- | --- | --- | --- | --- | --- | --- | --- |
|  |  |  |  |  |  |  |  |  |  |

FOCUS GROUP INTRODUCTION

WELCOME

Thanks for agreeing to be part of the focus group. We appreciate your willingness to participate.

PURPOSE OF FOCUS GROUPS

We have been asked by a research team from Arba Minch University to conduct the focus groups. The reason we are having these focus groups is to assess your attitude, experience and your communities’ view about MWHs

We need your input and want you to share your honest and open thoughts with us.

GROUND RULES

1. WE WANT YOU TO DO THE TALKING.

- We would like everyone to participate.
- I may call on you if I haven't heard from you in a while.

1. THERE ARE NO RIGHT OR WRONG ANSWERS

- Every person's experiences and opinions are important.
- Speak up whether you agree or disagree.
- We want to hear a wide range of opinions.

1. WHAT IS SAID IN THIS ROOM STAYS HERE

- We want folks to feel comfortable sharing when sensitive issues come up.

1. WE WILL BE TAPE RECORDING THE GROUP

- We want to capture everything you have to say.
- We don't identify anyone by name in our report. You will remain anonymous

Questions for a Focus Group

Let's start by going around the circle and having each person introduce herself.

1. Please tell me about the birth of last your baby.
   1. How satisfied were you with the setting? (Comfort, cleanliness, etc.)
   2. Who was with you? (Family, health care providers, etc.)
   3. How satisfied were you with the provider or providers?
2. How did this compare to your previous birth experiences, either your own or experiences you have heard from other women?
3. What do you think about the topic that has brought us here today (MWHs)?
4. In your opinion, what type of women would benefit from using the MWH?

PROBE FOR: Need based on health reasons, Need based on geography and Need based on socioeconomic factors

1. According to you, do you think that MWHs are good for pregnant women? Why? Can you provide examples please?
2. Some peoples say that if a mother decided to stay in MWHs, the rest children suffers a lot and therefore do not like to go there. Do you agree with their idea? Why? Would you explain by giving examples please?
3. What is your personal belief about MWHs?
4. Who did support you to stay in MWHs and how? Providers, husband and relatives
5. During the time you were living in the MWH, What things did you more enjoyed? What are the worst thinks there? What thinks do you recommend in order to improve the lives of the women at the MWHs in the future?
6. Some people say MWHs are source of infection? Do you agree? Why?
7. What more things can you say about the MWHs?
8. Do you have any questions?

*Thank you for taking the time to talk to us!!*
